# Optimization of polyethylene glycol-based isolation of exosomes from mesenchymal stem cells for regenerative medicine applications

**DOI:** 10.1186/s13287-026-05108-z

**Published:** 2026-06-15

**Authors:** Maryam Adelipour, Hyojin Hwang, Reham M. Marzouk, Mohamed A. Gab-Allah, Ga Seul Lee, Jeong Hee Moon, Kee K. Kim, David M. Lubman, Jeongkwon Kim

**Affiliations:** 1 Cellular and Molecular Research Center, Medical Basic Sciences Research Institute, Department of Biochemistry, Ahvaz Jundishapur University of Medical Sciences, Ahvaz, Iran; 2 Department of Chemistry, Chungnam National University, Daejeon, Republic of Korea; 3 Organic Analysis Laboratory, National Institute of Standards, Tersa St., Al-Haram, P. O. Box 136, Giza, 12211 Egypt; 4 Research Facility and Analysis Center, Korea Research Institute of Bioscience and Biotechnology, Daejeon, Republic of Korea; 5 Department of Biochemistry, Chungnam National University, Daejeon, Republic of Korea; 6 Department of Surgery, University of Michigan Medical Center, Ann Arbor, MI USA

**Keywords:** Mesenchymal stem cells, Exosome isolation, Polyethylene glycol, Proteomics, Metabolomics

## Abstract

**Background:**

Mesenchymal stem cells (MSCs) and their derived exosomes have gained significant attention in regenerative medicine due to their unique therapeutic properties, including immunomodulatory and regenerative capabilities. However, the development of a simple, scalable, and reproducible method for isolating exosomes from MSC-conditioned media remains a challenge.

**Methods:**

We optimized a polyethylene glycol (PEG)-based precipitation method for exosome isolation by initially evaluating three different PEG molecular weights (1140, 3350, and 8000 Da). Protein quantification and nanoparticle tracking analysis (NTA) were performed for all three PEG types to assess yield and particle size. Based on these results, PEG 3350 and PEG 8000 were selected for further characterization by scanning electron microscopy (SEM), size exclusion chromatography (SEC), and Western blotting. Subsequently, proteomic and metabolomic analyses were conducted using exosomes isolated with PEG 3350. For functional assays, HeLa cells were exposed to increasing concentrations of MSC-derived exosomes under either 1% or 10% fetal bovine serum (FBS). Cell viability was evaluated at 24 h and 48 h using the the 3-(4,5-dimethylthiazol-2-yl)-5-(3-carboxymethoxyphenyl)-2-(4-sulfophenyl)-2H-tetrazolium (MTS) assay.

**Results:**

Our results demonstrated that PEG 3350 provided the highest yield and purity of MSC-derived exosomes. The isolated exosomes exhibited an average size below 200 nm, as confirmed by SEM and NTA. Western blotting validated the presence of exosome-specific markers CD63, CD9, and LGALS3BP, while liquid chromatography–tandem mass spectrometry (LC-MS/MS) analysis identified 357 proteins and 1,085 metabolites, confirming the molecular integrity of the exosomes. Additionally, SEC analysis revealed that repeated PEG-based enrichment cycles effectively improved exosome purity by removing non-vesicular contaminants. Functionally, exosomes isolated with PEG 3350 exhibited no cytotoxicity in HeLa cells and, at higher concentrations, promoted a condition-dependent increase in cell viability.

**Conclusions:**

This study presents an optimized PEG 3350-based method for exosome isolation, providing a scalable and reproducible approach for obtaining high-purity MSC-derived exosomes. These findings have significant implications for the development of exosome-based therapeutics in regenerative medicine.

**Supplementary Information:**

The online version contains supplementary material available at https://doi.org/10.1186/s13287-026-05108-z.

## Background

Regenerative medicine aims to restore damaged tissues and organs by using advanced techniques to promote natural healing processes, offering hope for effective treatments of degenerative conditions [1, 2]. Mesenchymal stem cells (MSCs) have garnered attention in the field of regenerative medicine due to their unique properties, including self-renewal, differentiation capabilities, low immunogenicity, the ability to home to injured tissues, and antioxidative and anti-inflammatory capacities [3]. MSC-derived exosomes have been suggested to replicate the beneficial properties of MSCs—such as enhancing regeneration, modulating immunity, exhibiting anti-tumor effects, and penetrating barriers like the blood-brain barrier. Moreover, MSC-derived exosomes offer advantages over whole MSCs, including lack of tumorigenicity, reduced risk of immune rejection, nanoscale size that enhances administration efficiency, and superior biocompatibility compared to synthetic nanoparticles, further increasing interest in their use [4–6].

While the International Society for Extracellular Vesicles (ISEV) 2018 guidelines recommend the term “sEVs” to encompass the heterogeneity of small extracellular vesicles [7], we have chosen to use the term “exosomes” in this manuscript. This decision is based on the following considerations: (1) Our isolation method, which includes polyethylene glycol (PEG) precipitation followed by multiple enrichment cycles, is designed to specifically target vesicles within the expected size range and with the expected characteristics of exosomes (40–200 nm), as confirmed by nanoparticle tracking analysis (NTA) and electron microscopy (2). The term “exosomes” is still widely used in the literature, particularly in the context of MSC-derived vesicles, and using it allows for better clarity and consistency with existing research in this specific area (3). We have used exosome markers CD63, CD9, and galectin-3-binding protein (LGALS3BP) to characterize the isolated vesicles. We acknowledge the inherent challenges in definitively proving exosome origin without extensive proteomic analysis of specific marker combinations; however, based on the above criteria, the term “exosomes” is considered the most appropriate designation for this study.

Exosomes refer to small extracellular vesicles (sEVs) with a size range of 40–200 nm. These vesicles originate from the endosomal compartment and carry various biomolecules, such as proteins, nucleic acids, and lipids, performing a wide range of biological functions. Exosomes play a crucial role in both physiological and pathological processes by facilitating the transfer of materials and signaling between cells [8].

There are various methods for isolating exosomes based on factors such as density, size, and affinity [9]. One straightforward approach that does not require complex equipment is polymer-based isolation. This method is advantageous because it minimally affects the isolated exosomes and operates at neutral pH. However, it also allows for the co-isolation of non-vesicular contaminants, including lipoproteins, and the polymeric substances used may interfere with subsequent analyses [10]. Polyethylene glycol (PEG) is a widely used polymer for isolating exosomes; however, this isolation method necessitates optimization to accommodate the specific characteristics of various sample types [11]. As a step forward in the clinical application of MSC-derived exosomes, proteomics and metabolomics analyses could be beneficial. Understanding the protein content of exosomes derived from MSCs is essential for elucidating the potential mechanisms by which these exosomes affect host cells and their possible applications in the field of regenerative medicine. Liquid chromatography-tandem mass spectrometry (LC-MS/MS) is commonly used to detect low-abundance analytes [12]. MS-based proteomics is a powerful method for analyzing the majority of proteins present in a sample, allowing researchers to identify and quantify the proteins in exosomes, thereby providing insights into their functional roles and therapeutic potential [13]. Furthermore, metabolomics can offer valuable insights into the bioactive metabolites within MSC-derived exosomes and their implications in regenerative medicine. The application of LC-MS/MS in high-resolution MS provides an advanced analytical framework for profiling the metabolic content of exosomes [14]. This approach enables sensitive and quantitative analysis of a wide array of metabolites, allowing researchers to explore the functional roles of exosomes in intercellular communication and their potential therapeutic applications.

The objective of this study is to optimize and characterize a simple, accessible PEG-based method for isolating MSC-derived exosomes. Additionally, we aim to gain deeper insights into their molecular composition through proteomics and metabolomics analyses, advancing their potential use in regenerative medicine.

## Methods

### Exosome isolation from mesenchymal stem cells

**Fig. 1:**
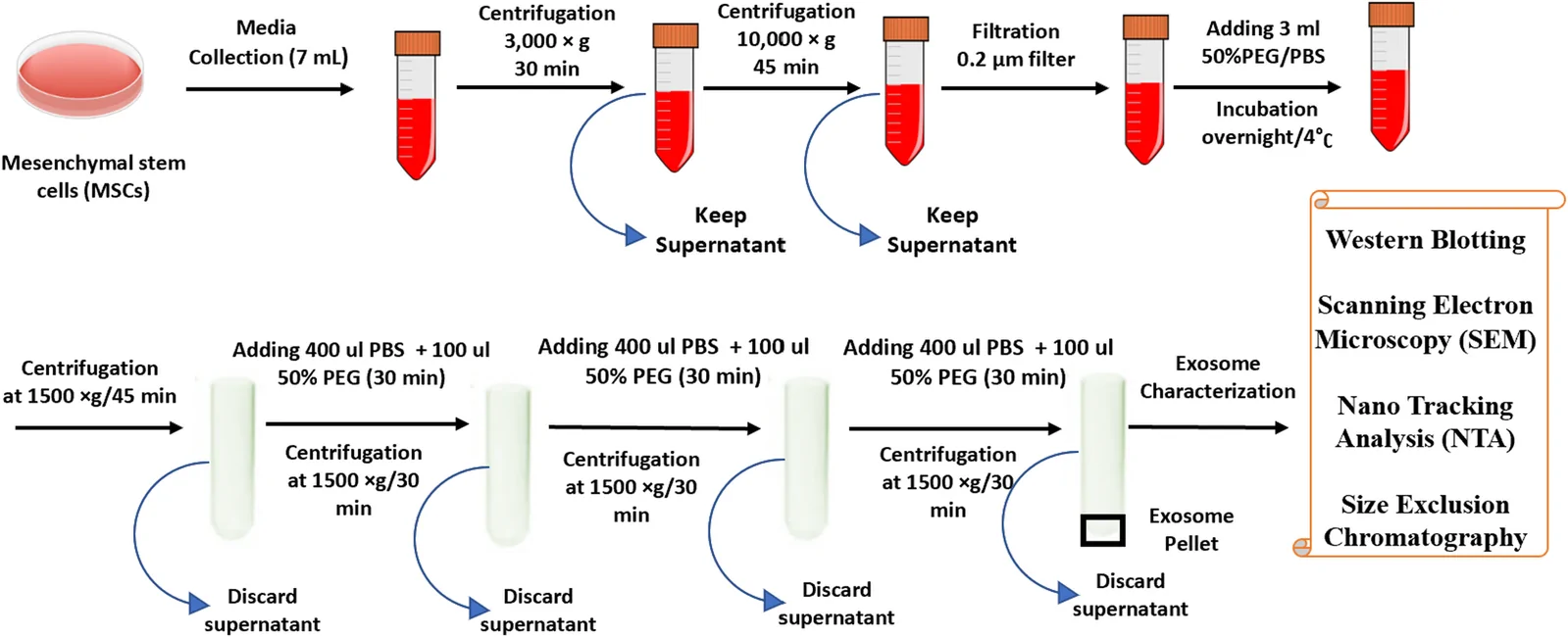
Exosome isolation from MSC conditioned media using PEG-mediated enrichment. MSCs were cultured in DMEM supplemented with FBS and Penicillin-Streptomycin, and the conditioned media was collected after 48 h. Exosomes were enriched through four cycles of PEG precipitation using three different molecular weights of PEG (1140, 3350, and 8000 Da). After each cycle, the supernatant was discarded, and the exosome pellet was resuspended and further enriched. The final exosome preparation was used for subsequent characterization

Human bone marrow-derived MSCs (PCS-500-012) were obtained from American Type Culture Collection (ATCC) and cultured in Dulbecco’s Modified Eagle Medium (DMEM) (Cat. LM001-11, Welgene, South Korea) supplemented with 10% fetal bovine serum (FBS) (Gibco, US) and 1% Penicillin-Streptomycin (Gibco, US) in 90 × 20 mm dishes (SPL, South Korea) under standard conditions of 37 °C, 5% CO_2_, and high humidity. Upon reaching 70% confluency, the media was replaced with serum-free media and incubated for 48 h. Then, the media was collected and centrifuged at 3000 × g for 30 min, followed by a second centrifugation of the supernatant at 10,000 × g for 45 min. The resulting supernatant was filtered through a 0.2 μm filter to obtain purified media (MSC-conditioned media). PEGs with molecular weights of 1140, 3350, and 8000 Da (PEG 1140, PEG 3350, and PEG 8000; Sigma-Aldrich, Saint Louis, MO, USA) were separately dissolved in Dulbecco’s phosphate-buffered saline (DPBS, referred to hereafter as PBS; Welgene, South Korea; Cat. No. LB001-02) to prepare 50% (wt/vol) PEG solutions. Each 3 mL PEG solution was mixed with 7 mL of purified media to create a 15% (wt/vol) PEG solution, which was incubated overnight at 4 °C. Centrifugation was performed at 1500 × g for 45 min at 4 °C, followed by removal of the supernatant and resuspension of the pellet in 70 µL of PBS, constituting the first cycle of exosome sample. For the second exosome enrichment, the exosome pellet from the first cycle was resuspended in 400 µL of PBS and 100 µL of 50% (wt/vol) PEG solution, incubated at 4 °C for 30 min, followed by centrifugation at 1500 × g for 30 min at 4 °C, after which the supernatant was removed, and the pellet was resuspended in 70 µL of PBS to constitute the second cycle of exosome sample. This step was repeated two more times for the third and fourth cycles of exosome samples (Fig. 1).

### Protein measurement of MSC-derived exosomes

Protein concentration of each enriched exosome sample was measured using the BCA protein assay according to the manufacturer’s instructions (ThermoFisher Scientific, Pierce™ BCA Protein Assay Kit). Briefly, 10 µL of each cycle exosome sample or standard was added to a 96-well plate, followed by 200 µL of working reagent, which was prepared by mixing reagent A and reagent B in a 50:1 ratio. Bovine serum albumin (BSA) standards were prepared in a concentration range of 25–1500 µg/mL. After thorough mixing, the plate was incubated at 37 °C for 30 min, then cooled to room temperature. The absorbance was measured at 562 nm using a microplate reader (SpectraMax ABS Plus^®^) located at CNU Chemistry Core Facility (Daejeon, South Korea). The blank value was subtracted from the absorbance values of all samples and standards. A standard curve was generated using linear regression, and the protein concentration of each sample (µg/µL) was calculated accordingly. The total protein amount (µg) was then determined by multiplying the exosome protein concentration (µg/µL) by the volume of each exosome sample (µL).

### Nanoparticle tracking analysis of MSC-derived exosomes

To assess the size of exosomes extracted from MSCs, each sample was diluted with PBS and subsequently analyzed using a NTA instrument, specifically the ZetaView (Particle Metrix, Germany). The dilution ratios ranged from 1:200 to 1:1000. For the NTA measurements, a blue laser with a wavelength of 488 nm was employed.

### Size exclusion chromatography of MSC-derived exosomes

A size exclusion chromatography (SEC) setup (CNU Chemi**s**try Core Facility, Daejeon, South Korea) was utilized with a 1000 Å Ultrahydrogel column (Waters, e2695), using PBS as the mobile phase. The column temperature was kept constant at 25 °C throughout the analysis. An injection volume of 10 µL was employed. Eluates were detected using a UV-visible detector (Waters, 2489), configured to the maximum wavelength (λmax). The flow rate was set at 1 mL/min, and the SEC system was allowed to equilibrate until the pressure fell below 450 psi prior to each sample injection. An equilibration time of 30 min was applied at the beginning of the analysis before running the samples. PBS was used as a blank between each sample, with each sample analyzed for 15 min.

### Scanning electron microscopy of MSC-derived exosomes

Scanning electron microscopy (SEM) (CNU Chemistry Core Facility, Daejeon, South Korea) from Seron (Gyeonggi-Do, South Korea) was utilized to analyze the morphology of exosomes. Sample preparation involved the following steps: 2 µL of each exosome sample (diluted 1:5 with distilled water) was placed onto a silicon wafer, followed by fixation with 2.5 µL of 4% glutaraldehyde. The samples were then dehydrated in a stepwise fashion using 25%, 50%, 75%, 90%, and 100% ethanol, with incubation at 37 °C overnight. Afterward, the samples were coated with gold (Au) for 120 s using an Ion Coater (SPT-20, Elim Global, Korea). SEM images were captured at 20.0 kV with magnifications ranging from 25,000× to 33,000×.

### Biomarker identification of MSC-derived exosomes using western blotting

Western blotting was performed to detect the presence of CD63, CD9, and LGALS3BP as positive exosome markers, and the absence of calnexin and calreticulin as negative markers. Exosome samples were lysed using RIPA buffer containing protease inhibitors, and 4 µg of protein from each sample (MSCs, filtered media, and exosome fractions) was subjected to SDS-PAGE on a 10% bisacrylamide/acrylamide gel. Electrophoresis was carried out for 15 min at 85 V, followed by 90 min at 150 V. Proteins were then transferred onto a polyvinylidene difluoride (PVDF) membrane using a semi-dry transfer cell at 10 V for 80 min (Bio-Rad, USA). The membrane was blocked with 5% skim milk for 60 min at room temperature, followed by overnight incubation at 4 °C with primary antibodies: anti-CD63 (ab59479, Abcam), anti-CD9 (10626D, Invitrogen), anti-LGALS3BP (ab67353, Abcam), anti**-**Calnexin (ab112995, Abcam), and anti-Calreticulin (ab22683, Abcam), each diluted 1:500 in PBST (0.1% Tween 20 in PBS). After three PBST washes, the membrane was incubated with secondary antibody, goat anti-mouse IgG H&L conjugated with HRP (ab97040, Abcam) at a 1:2000 dilution for 60 min. Following three additional washes, protein bands were visualized using an electrochemiluminescence (ECL) detection system with the Kwik Quant Western Blot Detection Kit (Kindle BioSciences, USA) and imaged using the Kwik Quant Pro instrument.

### Proteome analysis of MSC-derived exosomes

For the proteomic evaluation, the S-trap method was employed for sample preparation following the manufacturer’s guidelines. Initially, 150 µg of exosomal protein was digested into peptides using trypsin enzyme. The exosome samples were lysed with 5% sodium dodecyl sulfate (SDS) (Sigma-Aldrich, Japan) in 0.05 M triethylammonium bicarbonate (TEAB) (Merck, USA). This was followed by reduction using 120 mM tris(2-carboxyethyl) phosphine (TCEP) (Thermo Scientific, USA) at 55 °C for 15 min, and alkylation with iodoacetamide (Sigma, Germany) in 25 mM ammonium bicarbonate at room temperature for 10 min. The subsequent step involved acidification with 12% phosphoric acid, after which the samples were combined with a binding buffer of 0.1 M TEAB/MeOH and applied to an S-trap column. The column was then centrifuged at 4000 × g for 30 s, and the flow-through was discarded. The column was rinsed three times with a 0.1 M TEAB/MeOH solution. For digestion, 10 µg of trypsin dissolved in 125 mL of 50 mM TEAB was added to the column, which was then incubated at 47 °C for 2 h. The resulting peptides were eluted using a mixture of 50 mM ammonium bicarbonate, 0.2% formic acid, and 50% acetonitrile. Finally, peptide concentration was quantified using the Pierce™ quantitative peptide assay (Thermo Fisher, USA) as per the manufacturer’s instructions. In summary, 10 µL of each sample or standard was pipetted into a 96-well plate. This was followed by the addition of 180 µL of the working reagent, which was prepared by combining reagent A, reagent B, and reagent C in a ratio of 50:48:1. After mixing thoroughly, the plate was incubated at 37 °C for 15 min and then allowed to cool to room temperature. The absorbance was measured to generate a standard curve, which was used to calculate the peptide concentration of each sample (µg/µL) as described in the protein assay. Finally, the samples were dried and reconstituted in 0.1% formic acid at a peptide concentration of 1 µg/µL.

Proteomic profiling of exosomal peptides was performed using nano-LC-MS/MS on an RSLCnano U3000 system, equipped with a Double nanoViper™ PepMap™ C18 column (75 μm x 500 mm, 2 μm, P/N DNV75500PN) and connected to an Orbitrap Exploris 240 mass spectrometer (Thermo Fisher Scientific). To separate the peptides, solvent A was 0.1% formic acid in water, while solvent B was 0.1% formic acid in acetonitrile. The peptides were introduced at a flow rate of 300 nL/min. A 120-min gradient was applied for solvent B, starting with an initial composition of 2%, which increased to 20% within 10 min, then gradually rose to 32% over the next 20 min, and finally reached 95% before resetting. The nanospray source operated with a spray voltage of 1.9 kV and a capillary temperature of 275 °C. Full MS scans were conducted at a resolution of 90,000, an AGC target of 3 × 10^6^ ions, and a maximum injection time of 80 ms. The mass range scanned was m/z 350 to 1200. For MS/MS (MS^2^) acquisition, the 15 most intense ions in each cycle were selected for fragmentation using higher-energy collisional dissociation (HCD) at a normalized collision energy of 30%. These MS^2^ scans had a resolution of 30,000, an AGC target of 1 × 10^6^ ions, and a maximum injection time of 200 ms, with an isolation window of *m/z* 2. To optimize specificity, a dynamic exclusion of 20 s and an intensity threshold of 1 × 10^5^ ions were applied. Peptide identification was performed using MaxQuant software (Version: 2.6.4.0, Max-Planck Institute of Biochemistry). Protein expression levels were quantified using the label-free quantification (LFQ) method. Comparative analyses were conducted against the UniProt database for *Homo sapiens* (Human) (reference proteome ID: UP000005640; taxonomy ID: 9606; Reviewed (Swiss-Prot)), which was downloaded in September 2024. The proteomics analysis of MSCs was performed using the same method as for the MSC exosomes. A proteomics library was also generated for MSCs. To construct this library, digested peptides from MSC-extracted proteins were fractionated into 24 fractions and introduced into a nanoLC-MS/MS system. The raw data from these fractions were then processed as a library in MaxQuant, along with the raw data of MSC peptides, to enhance protein identification efficiency. A Venn diagram was created to compare the similarity between exosomal proteins and proteins from MSCs or exosomal proteins from a project in the ProteomeXchange repository (PXD020948), using the online Venn diagram tool (bioinformatics.psb.ugent.be/webtools/Venn). Cytoscape and the STRING plugin were used to visualize the network of exosomal proteins, while CluGO was employed for their functional analysis.

### Metabolome analysis of MSC-derived exosomes

Exosomal metabolites were analyzed using a Q Exactive Plus Liquid Chromatography-High Resolution Mass Spectrometry system (Thermo Fisher Scientific, US) at the CNU Chemistry Core Facility in Daejeon, South Korea. Initially, the samples were mixed thoroughly to ensure homogeneity. To precipitate proteins and remove them from the samples, three volumes of methanol were added to each sample. After vigorous shaking for 1 min, the samples were subjected to sonication for an additional 1 min to facilitate the extraction of metabolites. Subsequently, the samples were shaken again to ensure complete mixing. Following the extraction process, the samples were centrifuged at 22,600 × g for 15 min at 4 °C to separate the precipitated proteins from the supernatant. The supernatant was carefully collected and dried using a SpeedVac concentrator to eliminate solvents. The dried residues were reconstituted in a solvent mixture of 50% water, 50% acetonitrile (ACN), and 0.1% formic acid for LC separation using a Hypersil GOLD VANQUISH C18 UHPLC Column (100 × 2.1 mm, 1.9 μm). The flow rate was set at 0.3 mL/min. Solvent B was maintained at 5% for the first 0.5 min, increased to 20% at 2 min, and further to 60% at 14 min. It then increased to 95% at 16 min and was held for 2 min before decreasing to 5% at 19 min. The total run time was set to 20 min, with the column temperature maintained at 40 °C. The full mass scan range was set from 67 to 1000 m/z, with an AGC target of 3 × 10^6^ and a resolution of 70,000. Collision energy was set to 35. For MS², the AGC target was 1 × 10^6^, with a resolution of 17,500 and an isolation window of 2 *m/z*. The raw data were processed using Compound Discoverer (ThermoFisher Scientific, US) to identify compounds by matching them against a comprehensive database. Subsequently, MetaboAnalyst 6.0 was used for enrichment analysis based on the identified compound names. Only well-annotated compounds with exact or unique matches were mapped. The Small Molecule Pathway Database (SMPDB) was set as the reference library for pathway analysis. The metabolomics dataset and associated metadata have been deposited in the National Metabolomics Data Repository (NMDR) at the Metabolomics Workbench [15].

### MTS assay to evaluate the effects of MSC-derived exosomes on HeLa cell viability

HeLa cells were seeded at a density of 4,000 cells per well in 96-well flat-bottom plates (SPL, South Korea) in DMEM (Welgene, Cat. No. LM 001–11) supplemented with either 1% or 10% FBS. After overnight attachment, cells were treated with increasing concentrations of MSC-derived exosomes (2, 4, 8, or 10 µg exosomal protein per 200 µL medium). Untreated wells served as controls. Cell viability was assessed at 24 h and 48 h using the CellTiter 96^®^ AQueous One Solution Cell Proliferation Assay (MTS; Promega, Madison, WI, USA) according to the manufacturer’s instructions. Briefly, 20 µL of 3-(4,5-dimethylthiazol-2-yl)-5-(3-carboxymethoxyphenyl)-2-(4-sulfophenyl)-2 H-tetrazolium (MTS) assay reagent was added to each well and incubated for 1 h at 37 °C in a humidified incubator with 5% CO₂. Absorbance was measured at 490 nm using a microplate reader. Data were normalized to control wells (set to 1.0), and each condition was tested in triplicate with five technical replicates (*n* = 3 × 5 wells).

## Results

### Comparison of total protein yield in MSC-derived exosomes isolated with PEG of different molecular weights

**Fig. 2:**
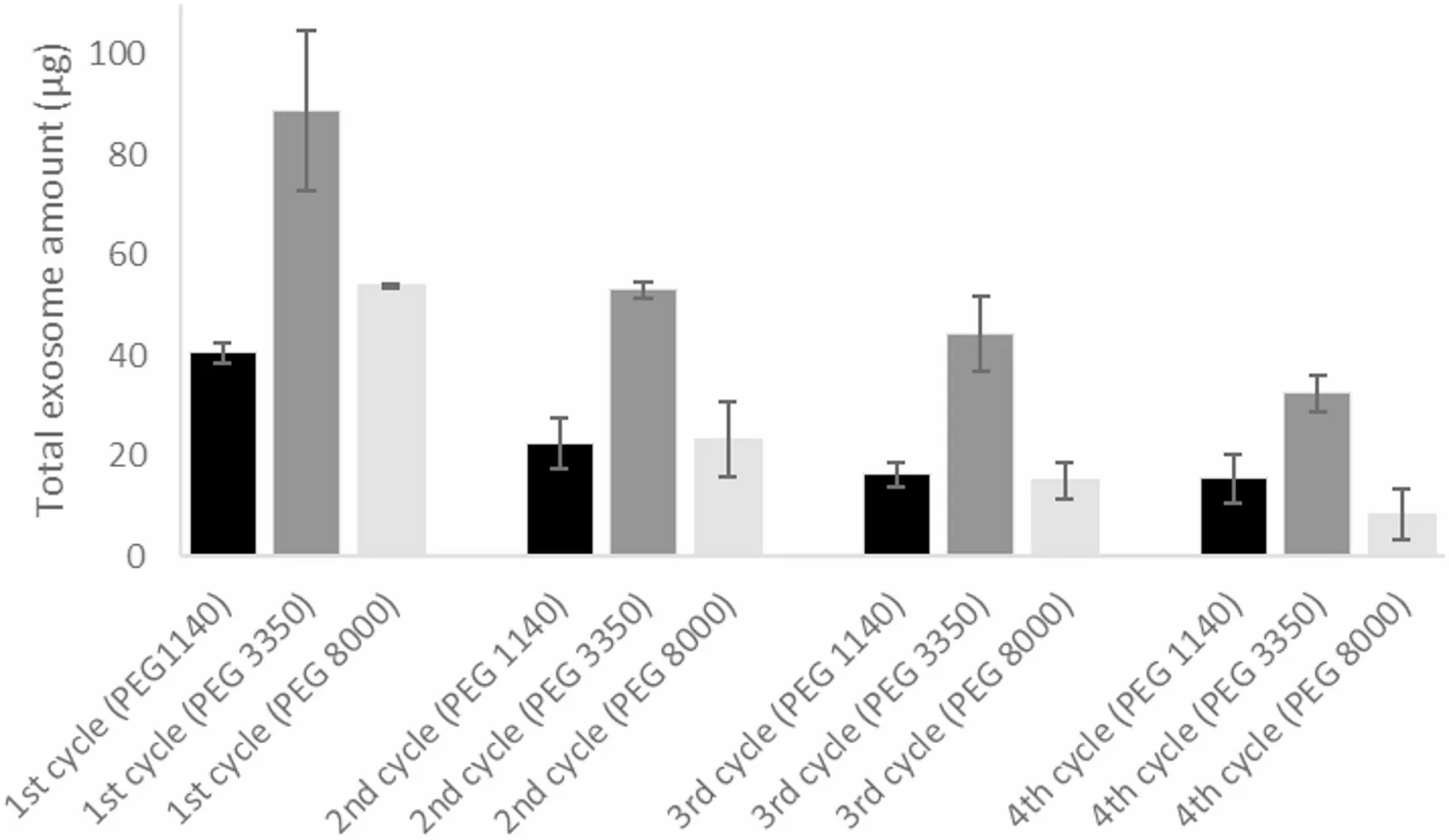
Total protein amount of exosomes derived from MSCs using three different PEGs of PEG 1140, PEG 3350, and PEG 8000. Standard deviation is displayed along with the average protein amount (*n* = 3). Comparing the four cycles of exosome enrichment, the first cycle yields the highest protein content, with protein amounts decreasing progressively in subsequent cycles. Additionally, PEG 3350 results in a higher protein yield compared to PEG 8000 or PEG 1140

The total protein amount in MSC-derived exosomes was measured after each enrichment cycle using three different PEGs of PEG 1140, PEG 3350 and PEG 8000 by calculating the exosome protein concentration (µg/µL) and multiplying it by the volume of each exosome sample (µL). As shown in Fig. 2, the total protein amount progressively decreases with each enrichment cycle. When comparing total exosome protein amounts across cycles, PEG 3350 consistently yields a higher amount than PEG 8000 or PEG 1140 per cycle. These findings highlight the potential of PEG 3350 for achieving optimal exosome recovery, which may improve the consistency and scalability of exosome isolation in regenerative medicine applications.

### Comparison of exosome size and concentration across enrichment cycles using different PEG molecular weights

**Fig. 3:**
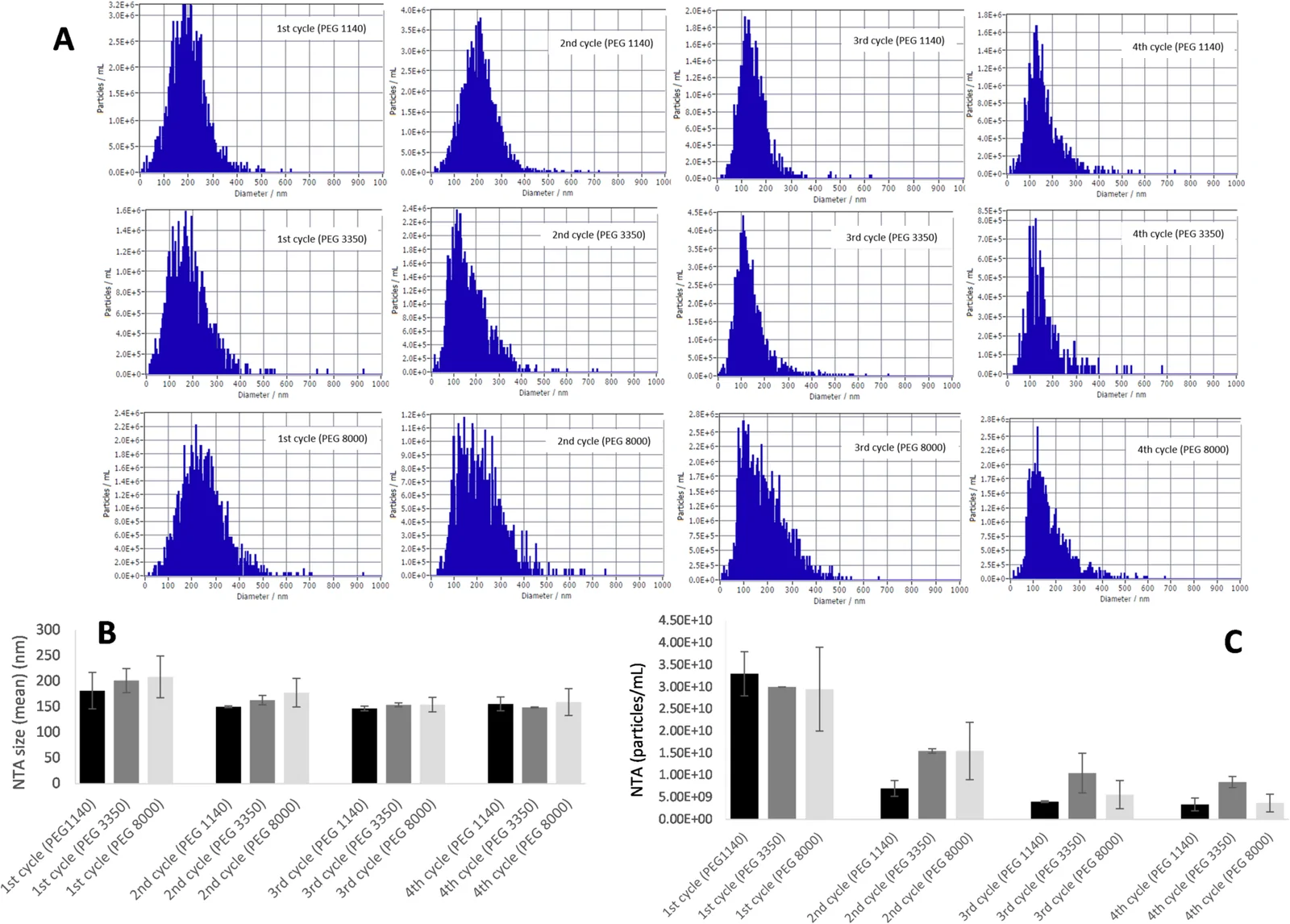
NTA analysis of exosomes derived from MSCs. **A** These graphs represent the results of exosomes extracted from MSCs after four cycles of isolation and enrichment using PEGs of three different molecular weights. **B** Size distribution of exosomes isolated using PEG with different molecular weights after each cycle of enrichment (4 cycles in total). **C** Concentration (particles/mL) of exosomes isolated using PEG with different molecular weights after each cycle of enrichment (4 cycles in total)

Figure 3A shows representative graphs of the size and concentration (particles/mL) of exosomes derived from MSCs, measured by NTA, obtained using PEGs of three different molecular weights across four enrichment cycles. As shown in Fig. 3B, when comparing particle sizes for each sample, exosomes extracted in the first cycle with PEG 3350 have an average size of 200 ± 23 nm, the second cycle 162 ± 9 nm, the third cycle 153 ± 3.6 nm, and the fourth cycle 148 ± 0.9 nm. For PEG 8000, the first cycle yields exosomes with an average size of 208 ± 40 nm, the second cycle 177 ± 27 nm, the third cycle 154 ± 14 nm, and the fourth cycle 159 ± 26 nm. For PEG 1140, the first cycle has an average size of 181 ± 35 nm, the second cycle 150 ± 1.6 nm, the third cycle 146 ± 4.5 nm, and the fourth cycle 155 ± 13 nm. The results indicate that, after the first cycle of exosome enrichment, the average size is less than 200 nm for all PEG molecular weights. Regarding particle concentration (particles per mL), as shown in Fig. 3C, a decline is observed from the first to the fourth cycle for all three PEG types. Notably, the average exosome size and concentration progressively decreased from the first to the fourth cycle for each PEG molecular weight. This trend suggests that repeated incubation with PBS containing PEG enhances exosome purity, likely due to the removal of aggregates of exosomes and proteins. To support this assumption, SEC demonstrates the removal of non-exosomal proteins after the first cycle, as explained in the following section. Since the particle concentration of exosomes isolated using PEG 1140 was significantly lower than that of exosomes isolated with PEG 3350 or PEG 8000 (Fig. 3C), further analyses were conducted only on the exosomes enriched with PEG 3350 and PEG 8000.

### SEC analysis of exosomes isolated from MSCs using PEG enrichment

**Fig. 4:**
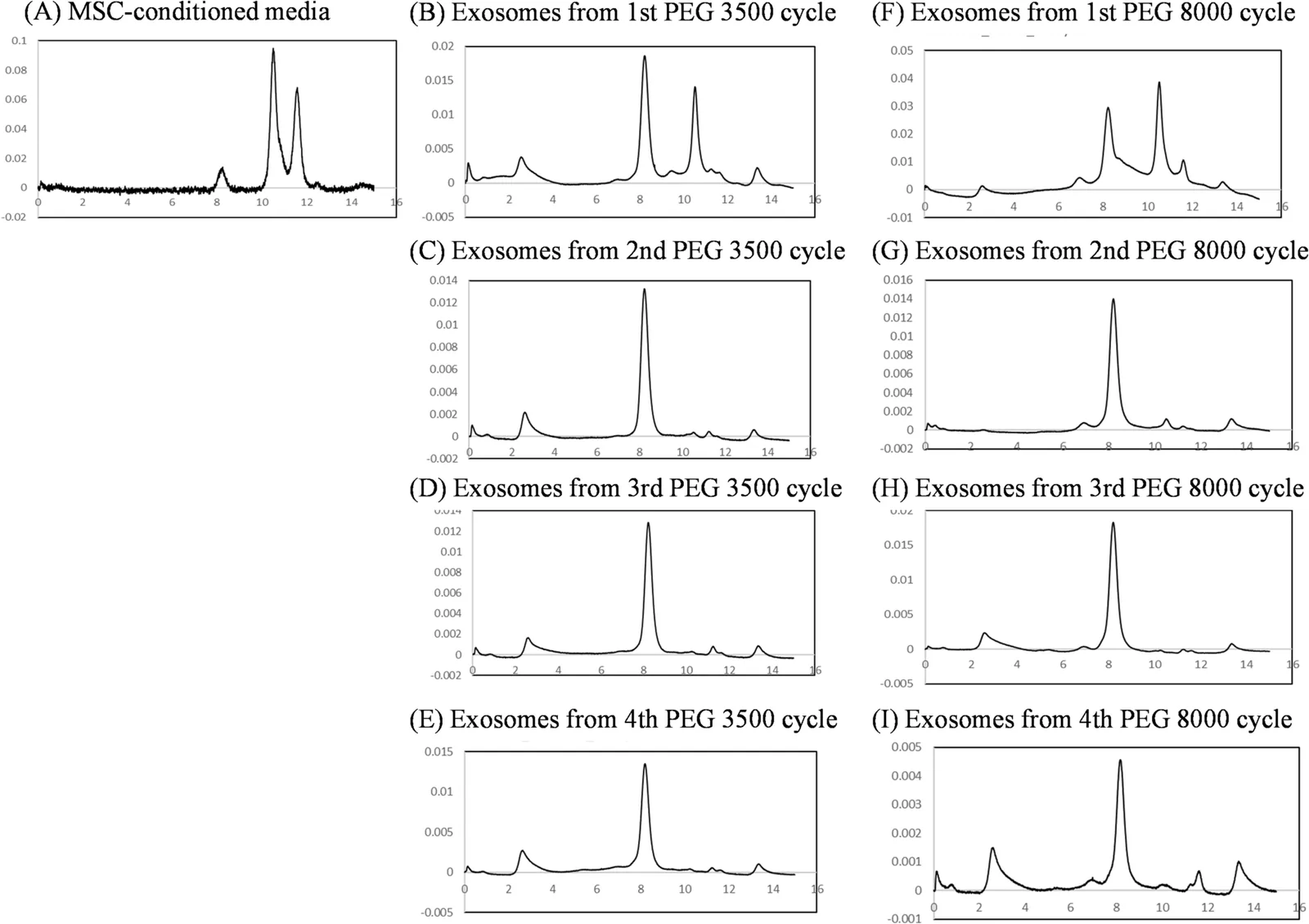
Size exclusion chromatograms of exosomes isolated from MSC-conditioned media using PEG 3500 and PEG 8000 across multiple precipitation cycles. **A** MSC-conditioned media before exosome isolation. **B**–**E** Exosomes isolated using PEG 3500 from the 1st to 4th precipitation cycles. **F**–**I** Exosomes isolated using PEG 8000 from the 1st to 4th precipitation cycles

SEC has been used to evaluate the efficiency of exosome isolation and enrichment from urine samples [16]. Here, exosomes isolated using PEG 3350 or PEG 8000 were analyzed via SEC. As shown in Fig. 4, MSC-conditioned media before PEG enrichment exhibited a small peak at 8 min and two larger peaks at 10.5 and 11.5 min. After the first cycle of PEG enrichment, the 8-min peak increased, while the 10.5-min and 11.5-min peaks significantly decreased. After the second cycle, the 10.5-min and 11.5-min peaks nearly disappeared, while the 8-min peak became dominant. This trend continued through the third and fourth cycles. Based on previous observations [16] and the current trend, the 8-min peak was considered to correspond to exosomes, while the 10.5-min and 11.5-min peaks likely represent proteins. The increase in the 8-min peak and the disappearance of the 10.5-min and 11.5-min peaks clearly demonstrate effective exosome enrichment using PEG 3350 and PEG 8000. Based on the chromatogram profiles in Fig. 4, PEG 3350 showed slightly better performance compared to PEG 8000.

### SEM characterization of exosome morphology after enrichment using PEG

**Fig. 5:**
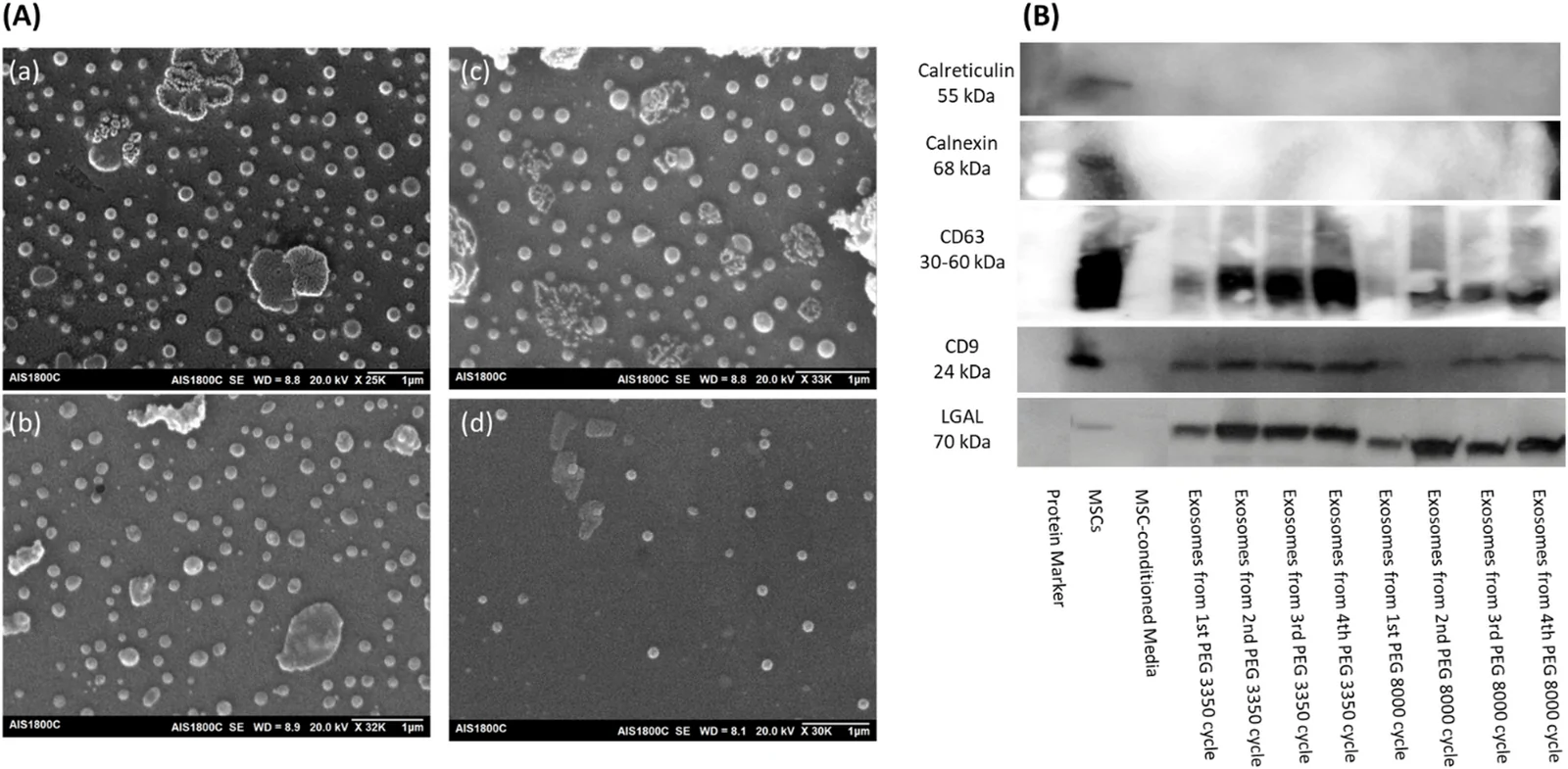
The results of the SEM and Western blot analysis of exosomes extracted from MSCs. **A** SEM images depict circular particles in the size range of less than 200 nm in the exosome fraction derived from MSCs using PEG 3350 Da: (a) 3rd cycle, (b) 4th cycle; and 8000 Da: (c) 3rd cycle, (d) 4th cycle. **B** Western blot analysis confirms the presence of CD63, CD9, and LGALS3BP as positive markers, and the absence of Calreticulin and Calnexin as negative markers in the exosome extracted from the media. Full-length blots are presented in Supplementary Figure S3

The exosome samples after the third and fourth cycles of enrichment using PEG 3350 or PEG 8000 were analyzed using SEM. The spherical shape of exosomes in each sample, within a size range of less than 200 nm, is shown in Fig. 5A. These images demonstrate the successful isolation and enrichment of exosomes, with consistent morphology observed across the different PEG molecular weights. In general, higher number of exosomes were observed from the enrichment using PEG 3350 than using PEG 8000.

### Western blot analysis of exosome markers

Exosomes extracted from MSC-conditioned media using PEG 3350 or PEG 8000 after each enrichment cycle, along with MSCs and MSC-conditioned media, were subjected to Western blot analysis to assess the expression of CD63, CD9, and LGALS3BP as positive markers of exosomes, and Calreticulin and Calnexin as negative markers. The sample of MSCs means the proteins extracted from MSCs using RIPA buffer. The sample of MSC-conditioned media means the culture media supposedly containing exosomes expressed from MSCs. As shown in Fig. 5B, both positive markers (CD63, CD9, and LGALS3BP) and negative markers (Calreticulin and Calnexin) were detected in MSCs, while none of them were observed in the MSC-conditioned media. After the first cycle of enrichment, the expression of the positive markers becomes detectable, with higher levels observed in subsequent cycles compared to the first cycle, indicating an increase in purity at the same protein amount (4 µg). According to established guidelines for exosome characterization, the detection of LGALS3BP suggests the binding of secreted proteins to the exosomes, further confirming their exosomal content [17]. When PEG 3350 and PEG 8000 were compared, the enrichment efficiency was higher using PEG 3350 than PEG 8000. SEC analysis, SEM analysis, and Western Blot Analysis all suggest that PEG 3350 is better than PEG 8000 in terms of exosome enrichment efficiency.

### Proteomic analysis of exosomes derived from MSCs

**Fig. 6:**
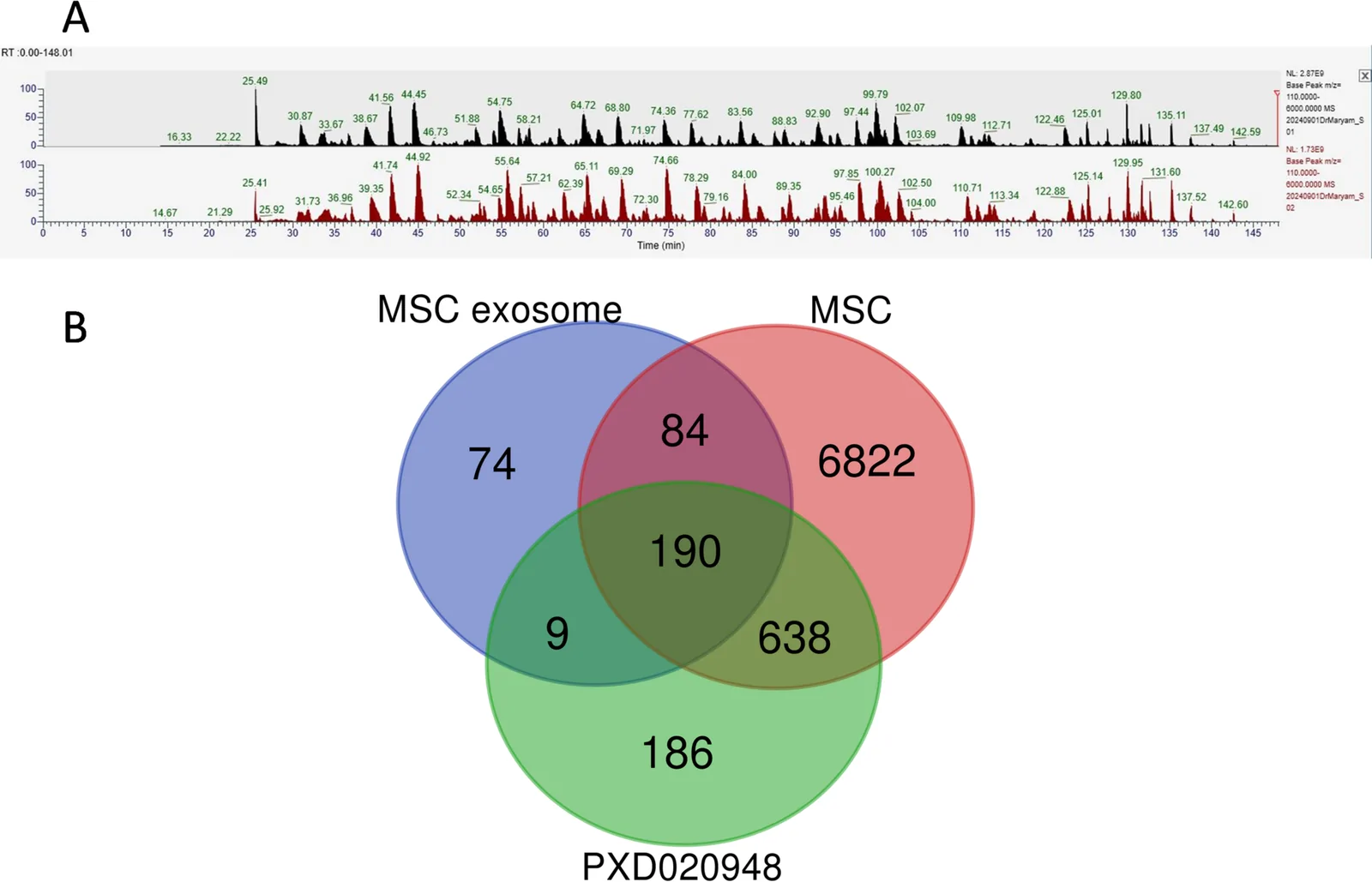
Proteomic profiling of exosomes derived from MSCs using nano-LC-MS/MS. **A** Chromatograms showing the peptide analysis of exosome proteins, and **B** Venn diagram illustrating the overlap of 357 currently identified proteins from the exosomes enriched with PEG 3350 (after three cycles), 7734 MSC proteins, and 1023 exosomes from a published project (PXD020948),

**Fig. 7:**
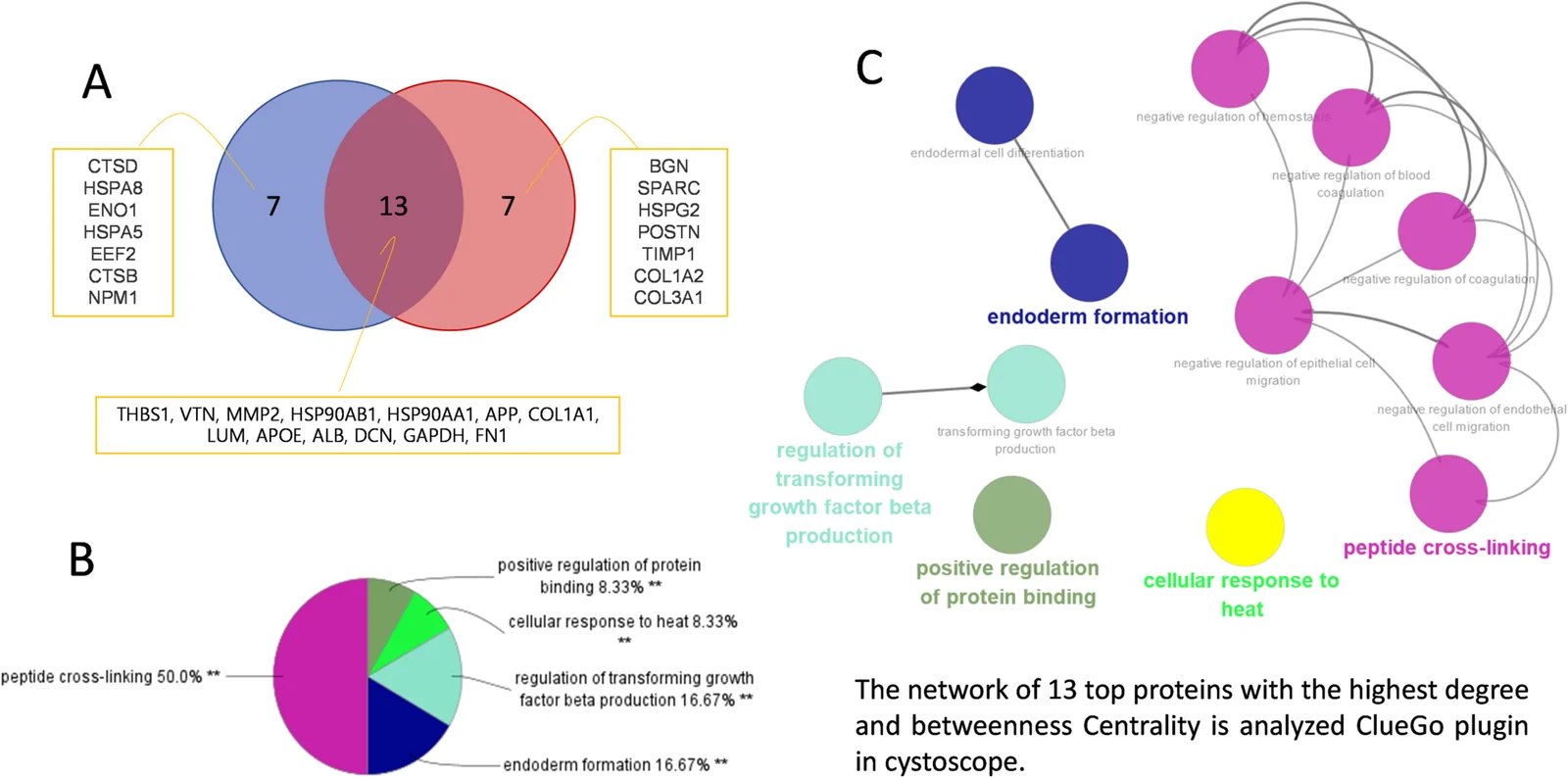
Network analysis of proteins with the highest betweenness centrality and degree. **A** Venn diagram illustrating the overlap of proteins with the highest betweenness centrality and degree, identifying the 13 proteins with both characteristics. **B** Pie chart depicting the molecular functions of these 13 proteins. **C** Network diagram illustrating the interactions between the 13 selected proteins

Peptides derived from exosome proteins, where the exosomes were enriched using a three-cycle PEG 3350 enrichment, were analyzed using nano-LC-MS/MS, as shown in the chromatograms (Fig. 6A). This analysis led to the identification of 357 proteins, including CD63 and LGALS3BP, which were also detected in the Western blot analysis of the exosome samples. Supplementary Figure S1 presents the MS/MS spectra of the peptides from CD63 and LGALS3BP. In a separate study, proteomics analysis of MSCs was performed, identifying 8,342 proteins, which, after removing duplicates and proteins without an exact gene name, resulted in a final dataset of 7,734 proteins. Figure 6B presents a Venn diagram illustrating the overlap among the 357 proteins identified in MSC-derived exosomes (MSC exosome) in this study, 7,734 MSC proteins (MSC) from a separate study deposited in the MassIVE repository (https://massive.ucsd.edu/) under dataset identifier MSV000094450, and 1,023 exosomal proteins from MSCs (PXD020948) reported in Wang’s study [18]. This overlap indicates that over 76% of the 357 identified proteins are consistent with their MSC origin. Additionally, 55% of the 357 proteins overlapped with previously published exosome protein profiles by Wang. In Wang’s published study, exosomes were isolated using an ultracentrifugation method that included gradient centrifugation at 300×g for 10 min, followed by 2000×g for 10 min, media filtration through a 0.45-µm porous membrane, and a final centrifugation at 120,000×g for 70 min [18]. The differences in protein profiles between this study and Wang’s may therefore be attributable to variations in isolation methodologies. Supplementary Figure S2 presents a network of all proteins detected in the exosomes in this study, constructed using Cytoscape with the STRING plugin to visualize protein-protein interactions, while their functional enrichment analysis, performed using CluGO, is provided in Supplementary Table S1. The network analysis of the identified proteins yielded 20 nodes with the highest betweenness centrality and 20 nodes with the highest degree. Figure 7A presents a Venn diagram illustrating the overlap between these two groups, highlighting the proteins with both the highest betweenness centrality and degree. Thirteen proteins were found to have both the highest betweenness centrality and degree, and their molecular functions are represented in a pie chart in Fig. 7B. Figure 7C shows the network of interactions among these 13 proteins. These 13 key proteins include Thrombospondin-1 (THBS1), Vitronectin (VTN), Matrix Metalloproteinase-2 (MMP2), Heat Shock Protein 90 Beta Family Member 1 (HSP90AB1), Heat Shock Protein 90 Alpha Family Class A Member 1 (HSP90AA1), Amyloid Precursor Protein (APP), Collagen Type I Alpha 1 Chain (COL1A1), Lumican (LUM), Apolipoprotein E (APOE), Albumin (ALB), Decorin (DCN), Glyceraldehyde-3-Phosphate Dehydrogenase (GAPDH), and Fibronectin 1 (FN1) and were enriched in functions related to cross-linking, which may impact blood hemostasis, coagulation, and cell migration. Additionally, some of these proteins are associated with endoderm formation and TGF-β response. Notably, HSP90 is known to be secreted via exosomes, as several studies have suggested that eHsp90α (extracellular Hsp90α) is secreted through this pathway [19]. However, further investigation is necessary to fully understand the roles of these exosomal proteins.

### Metabolomics analysis of exosomes derived from MSCs

**Fig. 8:**
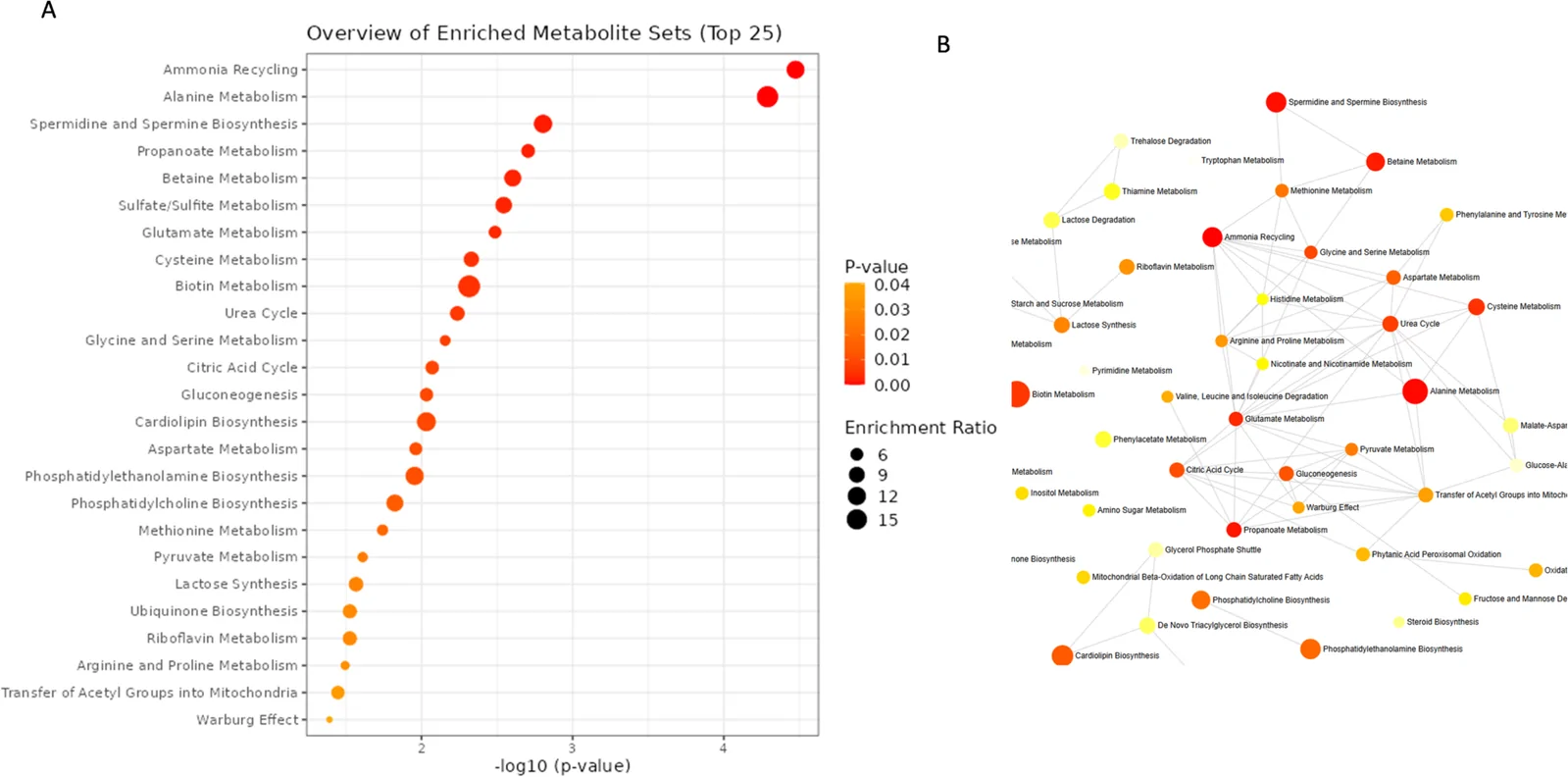
Metabolic enrichment analysis of exosome metabolites. **A** The top 25 pathways identified through enrichment analysis of 84 metabolites found in the HMDB pathway library. **B** Network diagram illustrating the interactions between the top 25 pathways

The metabolites from exosomes were extracted using methanol and analyzed using LC-MS/MS. A total of 1,085 metabolites were identified along with their MS/MS data as shown in Supplementary Table S2. Among the 1,085 metabolites, the enrichment analysis in MetaboAnalyst identified only 84 metabolites within the HMDB pathway library, corresponding to 78 pathways, which are listed in Supplementary Table S3. The top 25 of these pathways are shown in Fig. 8A. Figure 8B displays the network of interactions among the top 25 pathways. The metabolomics analysis of exosomes identified a broad range of metabolic pathways, highlighting their active role in cellular processes (Fig. 8). These included ammonia recycling and glutamate metabolism, both involved in nitrogen metabolism, and alanine metabolism, which plays a role in amino acid conversion. Spermidine and spermine biosynthesis highlighted polyamine production, while propanoate metabolism and betaine metabolism are associated with energy metabolism and methylation processes. Additionally, several pathways related to amino acid metabolism, including glutamate metabolism, cysteine metabolism, glycine and serine metabolism, and methionine metabolism, suggest that exosomes may support protein synthesis, antioxidant defense, and cellular signaling. Lipid biosynthesis was represented by cardiolipin and phosphatidylcholine biosynthesis. Supporting our findings, a recent study demonstrated that exosomes derived from primed mesenchymal stem cells (pMEX), exposed to hypoxia and serum-free conditions, contain a diverse array of primary metabolites and lipid molecules associated with key biosynthetic pathways, including amino acids, carbohydrates, and nucleosides. This study further showed that pMEX are enriched in ceramides and other lipid components essential for intercellular signaling and contain immunomodulatory metabolites such as glutamine and aspartic acid, which play roles in cellular communication and anti-inflammatory responses. Similarly, in our study, we used serum deprivation for 48 h. These findings indicate an almost identical metabolome in exosomes derived from MSCs [20].

### Effect of MSC-derived exosomes on HeLa cell viability

**Fig. 9:**
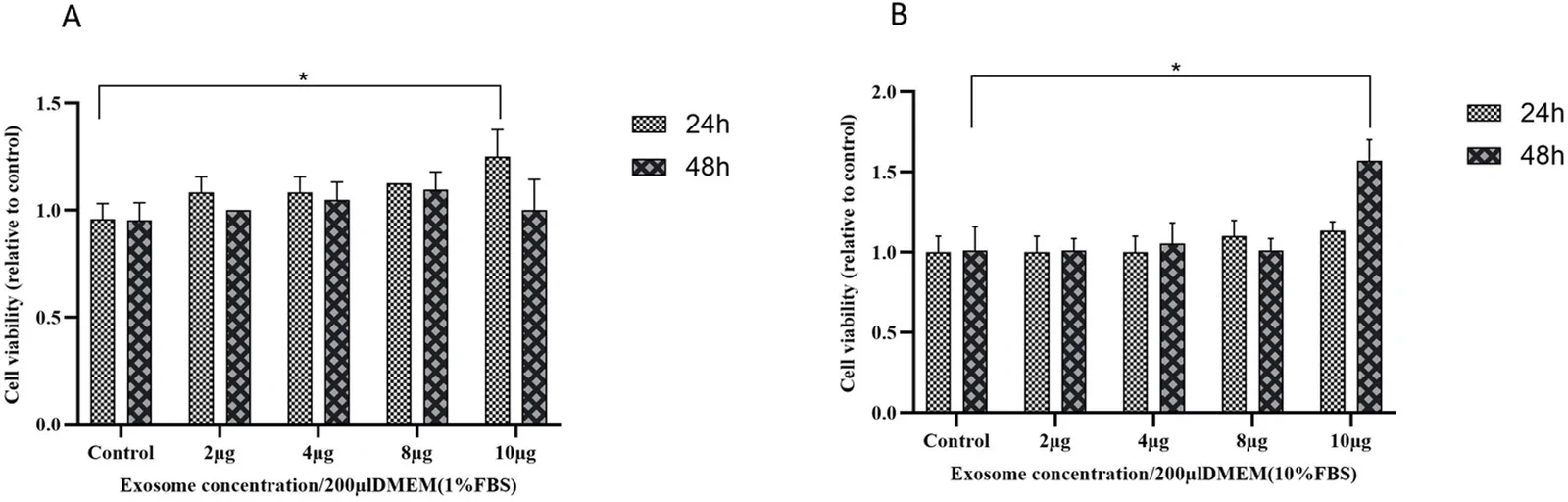
Effect of MSC-derived exosomes on HeLa cell viability assessed by MTS assay. **A** Viability of HeLa cells after 24 h and 48 h in 1% FBS with increasing exosome concentrations. **B** Viability of HeLa cells after 24 h and 48 h in 10% FBS with increasing exosome concentrations. Data represent mean ± SD from three independent experiments (*n* = 3 × 5 wells). A significant increase in viability was observed at the highest concentration (10 µg) after 24 h in 1% FBS and after 48 h in 10% FBS (*p < 0.05, one-way ANOVA with Dunnett’s post hoc test)

Treatment of HeLa cells with MSC-derived exosomes revealed no cytotoxic effects under any of the tested conditions and demonstrated a concentration- and condition-dependent influence on cell viability (Fig. 9). In 1% FBS medium, a significant increase in viability was observed after 24 h at the highest concentration (10 µg), while in 10% FBS medium, a similar increase became evident after 48 h. Lower concentrations of exosomes (2–8 µg) did not significantly alter cell viability compared with the control.

## Discussion

The optimization of exosome isolation from MSCs using PEG presents a significant advancement in the field of regenerative medicine. This study’s findings offer valuable insights into the development of a simple, accessible, and reproducible method for isolating MSC-derived exosomes, which could have far-reaching implications for therapeutic applications. Our results indicate that PEG 3350 provided exosome fractions with the highest yield and purity. This finding is crucial for researchers and clinicians seeking to isolate MSC-derived exosomes efficiently. The superior performance of PEG 3350 may be attributed to its optimal molecular weight, which strikes a balance between effective precipitation and minimal co-isolation of contaminants. Furthermore, the recovery rate of the optimized PEG method was comparable to that of commercial ExoQuick kits and significantly higher than ultracentrifugation, demonstrating its potential as a cost-effective and efficient alternative for exosome isolation [21].

The comprehensive characterization of isolated exosomes using various techniques strengthens the validity of our isolation method. SEM, NTA, and SEC consistently showed that the average size of the exosomes was less than 200 nm, confirming their expected size range. The spherical morphology observed under SEM further corroborates their exosomal nature. Western blotting confirmed the presence of CD63, CD9, and Galectin-3-binding protein (LGALS3BP) as positive markers, and the absence of Calnexin and Calreticulin as negative markers. This protein profile aligns with the established characteristics of exosomes, validating the specificity of our isolation method. Additionally, the use of multiple complementary techniques for exosome characterization, including electron microscopy, nanoparticle analysis, and western blotting, adheres to the guidelines suggested by the ISEV for comprehensive exosome verification [22].

The LC-MS/MS analysis of MSC-derived exosomes isolated using PEG 3350 revealed a rich molecular landscape. The identification of 357 proteins provides a comprehensive view of the exosomal proteome. This data set can serve as a valuable resource for future studies investigating the functional roles of specific proteins in exosome-mediated effects. The detection of 1,085 metabolites offers unprecedented insights into the metabolic cargo of MSC-derived exosomes. This metabolomic profile could be instrumental in understanding the bioactive components that contribute to the therapeutic potential of these exosomes. Furthermore, the comprehensive characterization of the exosomal proteome and metabolome aligns with recent advancements in the field, as similar studies have demonstrated that MSC-derived exosomes are packaged with numerous primary metabolites clustered in metabolic networks associated with amino acids, carbohydrates, and nucleoside biosynthetic pathways [20].

Importantly, our functional assays confirmed that exosomes isolated with PEG 3350 exhibited no cytotoxicity toward HeLa cells under either low- or high-serum conditions. At lower concentrations (2–8 µg), cell viability remained comparable to controls, while higher concentrations (10 µg) promoted a significant, condition-dependent increase in viability—after 24 h in 1% FBS and after 48 h in 10% FBS. This temporal difference can be explained by the culture environment. In serum-deprived conditions (1% FBS), HeLa cells are subjected to metabolic stress and rapidly rely on external support, enabling the pro-survival activity of exosomes to appear earlier. In contrast, under nutrient-rich conditions (10% FBS), endogenous growth factors initially maintain cell viability, and the supplementary effects of exosomal cargo become evident only after extended incubation.

These results are consistent with previous reports showing that MSC-derived exosomes can support cancer cell survival by enhancing vascular endothelial growth factor (VEGF) expression through activation of the extracellular signal-regulated kinase 1/2 (ERK1/2) pathway [23]. However, exosomes are known to exert a dual role in cancer, with both tumor-promoting and inhibitory effects reported depending on context [24]. The MTS assay demonstrated that at low doses, MSC-derived exosomes were safe for HeLa cells and exhibited no intrinsic cytotoxicity, highlighting their potential as drug delivery vehicles. The absence of any toxic effects further underscores the biosafety of exosomes isolated by this PEG-based method, supporting their suitability for downstream therapeutic applications.

The preserved bioactivity and non-cytotoxic behavior of the isolated exosomes confirm that they remain therapeutically competent—an essential requirement for their application in regenerative medicine. MSC-derived exosomes function as key paracrine mediators that recapitulate many of the therapeutic effects of their parent MSCs. They carry bioactive cargo, including growth factors, signaling lipids, mRNAs, and regulatory miRNAs, which modulate immune responses, reduce inflammation, and activate endogenous repair pathways [25]. Exosomes derived from placental MSCs directly stimulate angiogenesis by enhancing endothelial cell migration, tube formation, and angiogenesis-associated gene expression, contributing to microvascular regeneration in ischemic tissues [26]. In particular, MSC-derived exosomes have demonstrated therapeutic benefits in models of cardiac ischemia and myocardial infarction, where they exert anti-inflammatory, anti-apoptotic, pro-angiogenic, and anti-fibrotic effects that support functional cardiac recovery [27]. MSC-derived exosomes also exert potent immunomodulatory effects in chronic inflammatory diseases. In rheumatoid arthritis, MSC-derived extracellular vesicles suppress pro-inflammatory cytokine release, inhibit effector T cell activation, and shift macrophage polarization toward a reparative M2 phenotype, thereby reducing synovial inflammation and preserving joint structure [28]. Beyond cardiac and inflammatory diseases, MSC-derived exosomes have also shown regenerative potential in osteoarthritis, where they deliver regulatory RNAs, proteins, and lipid mediators that enhance chondrocyte survival and rebalance extracellular matrix turnover, thereby slowing cartilage degeneration and promoting structural repair [29]. Importantly, MSC-derived exosomes are already progressing toward clinical translation. Several ongoing and completed clinical trials have evaluated MSC-derived exosomes for applications ranging from immune modulation to tissue repair, highlighting their advantages over cell-based MSC therapies, including reduced infusion-related toxicity, lower tumorigenic risk, and improved storage stability [30].

The optimized PEG-based isolation method developed in this study offers several advantages for regenerative medicine applications. Since the clinical translation of MSC-derived exosomes requires scalable, reproducible, and Good Manufacturing Practice (GMP)-compliant isolation workflows [17], the simplicity and accessibility of PEG precipitation make this approach suitable for large-scale production of exosomes. Importantly, the optimized PEG 3350 workflow achieved high-yield recovery while preserving membrane integrity and bioactive cargo, indicating that the gentle nature of PEG-based isolation supports the retention of exosome functionality—an essential factor for maintaining their therapeutic efficacy in cell-free regenerative medicine. The consistent characterization results obtained across analytical platforms further demonstrate the high reproducibility of this method, which is crucial for standardization in clinical manufacturing settings. Previous studies have also shown that PEG-based approaches can isolate exosomes efficiently while maintaining cargo composition, and can significantly enhance exosome yield and purity, reinforcing the translational potential of this strategy [31]. Collectively, these features position the optimized PEG 3350 method as a promising platform for scalable, reproducible, and GMP-compatible production of clinical-grade MSC-derived exosomes.

While this study provides a solid foundation for PEG-based isolation of MSC-derived exosomes, several avenues for future research emerge. Investigating the biological activities of these isolated exosomes in various disease models would further validate their therapeutic potential [32, 33]. Comparing the efficiency and purity of this PEG-based method with other isolation techniques could help establish its relative advantages and limitations. The well-characterized exosomes obtained through this method could serve as a platform for exosome engineering, potentially enhancing their therapeutic efficacy. In summary, this optimized PEG-based isolation method for MSC-derived exosomes, coupled with comprehensive proteomic and metabolomic analyses, represents a significant step forward in harnessing the potential of exosomes for regenerative medicine. The simplicity, reproducibility, and effectiveness of this approach pave the way for accelerated research and development in exosome-based therapies. Building on these findings, future investigations should directly assess the functional regenerative effects of PEG-isolated MSC-derived exosomes in disease models relevant to tissue repair, such as endothelial wound healing, myocardial ischemia/reperfusion injury, and fibrosis attenuation. Additionally, evaluating dose–response, delivery routes, and therapeutic durability will be essential for advancing these exosomes toward clinical translation. These investigations will help establish whether the optimized PEG-based workflow can reliably support exosome-mediated angiogenic, immunomodulatory, and reparative outcomes in vivo.

## Conclusion

In this study, we demonstrated that PEG precipitation is an effective, accessible, and reproducible method for isolating exosomes from MSC-conditioned media. Exosomes were successfully isolated using three different molecular weights of PEG: 1140 Da, 3350 Da, and 8000 Da, with PEG 3350 proving to be the most effective, providing an optimal balance of purity and yield. Notably, we found that achieving high purity requires multiple enrichment cycles; we recommend at least two cycles to ensure the isolation of high-quality exosomes. Since PEG facilitates precipitation by absorbing water, the high-water content of MSC-conditioned media necessitates a relatively high PEG concentration to maximize yield. Additionally, this study identified 357 proteins and 1,085 metabolites in MSC-derived exosomes isolated using PEG 3350. However, further research is needed to better understand the characteristics and therapeutic potential of MSC-derived exosomes, which could pave the way for their application in regenerative medicine and other therapeutic fields. Importantly, functional assays confirmed that PEG 3350–isolated exosomes exhibited no cytotoxicity in HeLa cells and even promoted a condition-dependent increase in viability at higher concentrations, supporting their biosafety and potential utility in therapeutic applications. However, further research is needed to better understand the characteristics and therapeutic potential of MSC-derived exosomes, which could pave the way for their application in regenerative medicine and other therapeutic fields.

## Supplementary Information

Supplementary Material. Figure S1. MS/MS spectra confirming the identification of CD63 and LGALS3BP (Galectin-3–binding protein) in MSC-derived exosomes isolated using the optimized PEG 3350 method. Panel (A1) shows the fragmentation spectrum of a representative peptide from CD63. Panels (B1–B19) display MS/MS spectra for multiple unique peptides mapped to LGALS3BP, confirming its high-confidence identification. For each peptide, the theoretical monoisotopic mass-to-charge ratio (MH⁺) and any post-translational or preparation-related modifications (e.g., methionine oxidation, cysteine carbamidomethylation) are indicated. These spectra support the proteomic findings shown in Fig. 6 and validate the successful enrichment of exosomal marker proteins in the isolated vesicle fraction. Figure S2. Protein–protein interaction (PPI) network of the 357 proteins identified in MSC-derived exosomes. The network was constructed in Cytoscape using the STRING database to visualize experimentally supported and predicted protein–protein interactions. Node color represents node degree on a continuous scale, with purple indicating the highest connectivity and yellow indicating the lowest connectivity. Network parameters for each protein—including degree, betweenness centrality, clustering coefficient, and average shortest path length—are provided in Supplementary Table S1. These parameters were used to identify the top 20 high-degree and top 20 high-betweenness proteins. The 13 proteins shared between these two groups were selected as key hub proteins. Functional enrichment of these 13 hub proteins was performed using the CluGO plugin in Cytoscape, and the resulting functional clusters and interaction maps are shown in Fig. 7. Figure S3. Full-length western blots for Calreticulin, Calnexin, CD63, CD9, and LGALS3BP, corresponding to the images in Fig. 5(B). Table S1. Network analysis metrics of MSC-derived exosomal proteins identified by LC–MS/MS. Table S2. List of metabolites identified in MSC-derived exosomes by LC–MS/MS. Table S3. Pathway enrichment analysis of metabolites identified in MSC-derived exosomes.

## Data Availability

The data supporting the findings of this study are available from the corresponding author upon request. The proteomics and metabolomics datasets generated during the current study are available in public repositories. The raw proteomics data and associated metadata are accessible via the MassIVE repository (ID: MSV000097756) at http://MSV000097756@massive-ftp.ucsd.edu. The metabolomics data have been deposited in the National Metabolomics Data Repository (NMDR) at the Metabolomics Workbench (Project ID: PR002880, Study ID: ST004570) and can be accessed via the DOI: https://doi.org/10.21228/M8D56M.

## Abbreviations

MSCs: Mesenchymal stem cells
PEG: Polyethylene glycol
SEM: Scanning electron microscopy
NTA: Nanoparticle tracking analysis
SEC: Size exclusion chromatography
LC-MS/MS: Liquid chromatography-tandem mass spectrometry
LGALS3BP: Galectin-3-binding protein
sEVs: Small extracellular vesicles
ISEV: International Society for Extracellular Vesicles
DMEM: Dulbecco’s Modified Eagle Medium
FBS: Fetal bovine serum
PBS: Phosphate-buffered saline
BSA: Bovine serum albumin
SDS-PAGE: Sodium Dodecyl Sulfate-Polyacrylamide Gel Electrophoresis
PVDF: Polyvinylidene Difluoride
PBST: Phosphate-buffered saline with Tween 20
HRP: Horseradish Peroxidase
ECL: Electrochemiluminescence
TEAB: Triethylammonium bicarbonate
TCEP: Tris(2-carboxyethyl) phosphine
