# Supplementary material for: Optimization of polyethylene glycol-based isolation of exosomes from mesenchymal stem cells for regenerative medicine applications
Source: Stem Cell Res Ther. 2026 Jun 15;17:316. doi: 10.1186/s13287-026-05108-z (PMC13579858; doi:10.1186/s13287-026-05108-z)
Supplement: Supplementary file 1 [file 13287_2026_5108_MOESM1_ESM.zip › New folder/Supplementary_Figure_S1_Network_02.pdf]

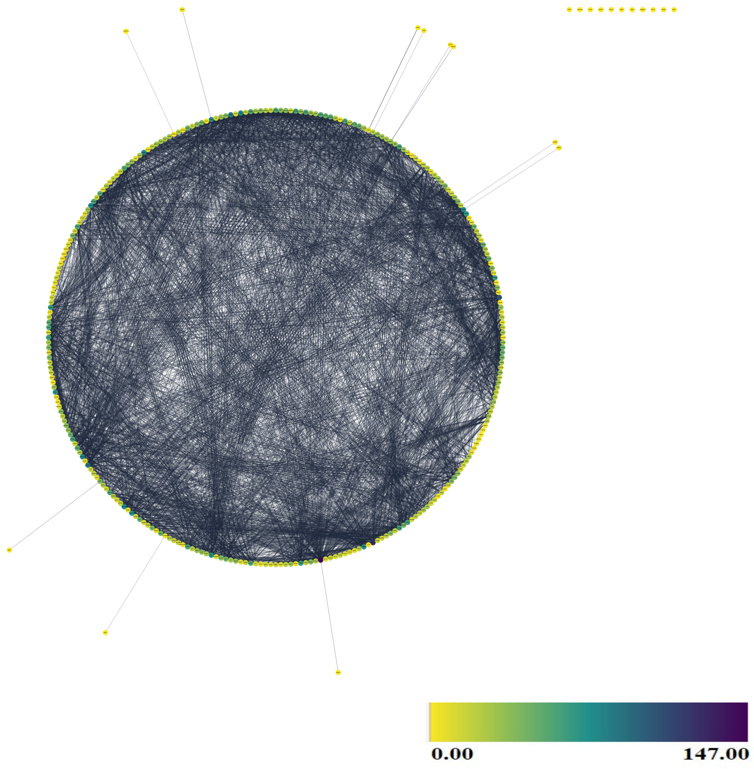

Supplementary Figure S1. Protein–protein interaction (PPI) network of the 357 proteins identified in MSC-derived exosomes. The network was constructed in Cytoscape using the STRING database to visualize experimentally supported and predicted protein–protein interactions. Node color represents node degree on a continuous scale, with purple indicating the highest connectivity and yellow indicating the lowest connectivity. Network parameters for each protein—including degree, betweenness centrality, clustering coefficient, and average shortest path length—are provided in Supplementary Table S1. These parameters were used to identify the top 20 high-degree and top 20 high-betweenness proteins. The 13 proteins shared between these two groups were selected as key hub proteins. Functional enrichment of these 13 hub proteins was performed using the CluGO plugin in Cytoscape, and the resulting functional clusters and interaction maps are shown in Figure 7.
