# Supplementary material for: Optimization of polyethylene glycol-based isolation of exosomes from mesenchymal stem cells for regenerative medicine applications
Source: Stem Cell Res Ther. 2026 Jun 15;17:316. doi: 10.1186/s13287-026-05108-z (PMC13579858; doi:10.1186/s13287-026-05108-z)
Supplement: Supplementary file 1 [file 13287_2026_5108_MOESM1_ESM.zip › New folder/Supplementary_Figure_S2_MSMS-spectra_02.docx]

**Supplementary Figure S2.** MS/MS spectra confirming the identification of CD63 and LGALS3BP (Galectin-3–binding protein) in MSC-derived exosomes isolated using the optimized PEG 3350 method. Panel (A1) shows the fragmentation spectrum of a representative peptide from CD63. Panels (B1–B19) display MS/MS spectra for multiple unique peptides mapped to LGALS3BP, confirming its high-confidence identification. For each peptide, the theoretical monoisotopic mass-to-charge ratio (MH⁺) and any post-translational or preparation-related modifications (e.g., methionine oxidation, cysteine carbamidomethylation) are indicated. These spectra support the proteomic findings shown in Figure 6 and validate the successful enrichment of exosomal marker proteins in the isolated vesicle fraction.

(A-1) CD63 (P08962, CD63_HUMAN); sequence VMSEFNNNFR w/1xOxidation; Theo. MH+ [Da] 1273.563

(B-1) LGALS3BP (Q08380 · LG3BP_HUMAN); sequence AAFGQGSGPIMLDEVQCTGTEASLADCK w/2xCarbamidomethyl [C17; C27]; 1xOxidation [M11]; Theo. MH+ [Da] 2929.30123

(B-2) LGALS3BP (Q08380 · LG3BP_HUMAN); sequence TLQALEFHTVPFQLLAR; Theo. MH+ [Da] 1984.10146

(B-3) LGALS3BP (Q08380 · LG3BP_HUMAN); sequence STSSFPCPAGHFNGFR w/1xCarbamidomethyl [C7]; Theo. MH+ [Da] 1768.78601

(B-4) LGALS3BP (Q08380 · LG3BP_HUMAN); sequence STHTLDLSR; Theo. MH+ [Da] 1029.53235

(B-5) LGALS3BP (Q08380 · LG3BP_HUMAN); sequence SQLVYQSR; Theo. MH+ [Da] 980.51597

(B-6) LGALS3BP (Q08380 · LG3BP_HUMAN); sequence SDLAVPSELALLK; Theo. MH+ [Da] 1355.77806

(B-7) LGALS3BP (Q08380 · LG3BP_HUMAN); sequence RIDITLSSVK; Theo. MH+ [Da] 1131.6732

(B-8) LGALS3BP (Q08380 · LG3BP_HUMAN); sequence IYTSPTWSAFVTDSSWSAR; Theo. MH+ [Da] 2162.01891

(B-9) LGALS3BP (Q08380 · LG3BP_HUMAN); sequence YSSDYFQAPSDYR; Theo. MH+ [Da] 1598.67578

(B-10) LGALS3BP (Q08380 · LG3BP_HUMAN); sequence IDITLSSVK; Theo. MH+ [Da] 975.57209

(B-11) LGALS3BP (Q08380 · LG3BP_HUMAN); sequence LADGGATNQGR; Theo. MH+ [Da] 1059.51776

(B-12) LGALS3BP (Q08380 · LG3BP_HUMAN); sequence KSQLVYQSR; Theo. MH+ [Da] 1108.61093

(B-13) LGALS3BP (Q08380 · LG3BP_HUMAN); sequence GQWGTVCDNLWDLTDASVVCR w/ 2xCarbamidomethyl [C7; C20]; Theo. MH+ [Da] 2452.102

(B-14) LGALS3BP (Q08380 · LG3BP_HUMAN); sequence ELSEALGQIFDSQR; Theo. MH+ [Da] 1592.79148

(B-15) LGALS3BP (Q08380 · LG3BP_HUMAN); sequence AVDTWSWGER; Theo. MH+ [Da] 1206.55381

(B-16) LGALS3BP (Q08380 · LG3BP_HUMAN); sequence ASHEEVEGLVEK; Theo. MH+ [Da] 1326.65359

(B-17) LGALS3BP (Q08380 · LG3BP_HUMAN); sequence ALMLCEGLFVADVTDFEGWK w/ 1xCarbamidomethyl [C5]; 1xOxidation [M3]; Theo. MH+ [Da] 2317.08792

(B-18) LGALS3BP (Q08380 · LG3BP_HUMAN); sequence LASAYGAR; Theo. MH+ [Da] 808.43118

(B-19) LGALS3BP (Q08380 · LG3BP_HUMAN); sequence YYPYQSFQTPQHPSFLFQDK; Theo. MH+ [Da] 2521.18229
