# Supplementary material for: Optimization of polyethylene glycol-based isolation of exosomes from mesenchymal stem cells for regenerative medicine applications
Source: Stem Cell Res Ther. 2026 Jun 15;17:316. doi: 10.1186/s13287-026-05108-z (PMC13579858; doi:10.1186/s13287-026-05108-z)
Supplement: Supplementary file 1 [file 13287_2026_5108_MOESM1_ESM.zip › New folder/Supplementary_Figure_S3_Original-blot-images_01.docx]

Supplementary Figure S3

**Supplementary Figure S3.** Full-length western blots for Calreticulin, Calnexin, CD63, CD9, and LGALS3BP, corresponding to the images in Figure 5(B).
